# Does adaptive radiotherapy for head and neck cancer favorably impact dosimetric, clinical, and toxicity outcomes?: A review

**DOI:** 10.1097/MD.0000000000038529

**Published:** 2024-06-28

**Authors:** Foteini Simopoulou, George Kyrgias, Ioannis Georgakopoulos, Rafaela Avgousti, Christina Armpilia, Pantelis Skarlos, Vasiliki Softa, Kiki Theodorou, Vassilis Kouloulias, Anna Zygogianni

**Affiliations:** a Radiation Oncology Unit, 1st Department of Radiology, Aretaieion University Hospital, Medical School, National and Kapodistrian University of Athens (NKUOA), Athens, Greece; b Radiation Oncology Department, University Hospital of Larissa, Faculty of Medicine, School of Health Sciences, University of Thessaly, Larissa, Greece; c Medical Physics Department, University Hospital of Larissa, Faculty of Medicine, School of Health Sciences, University of Thessaly, Larissa, Greece; d Radiation Oncology Department, Metropolitan Hospital, Piraeus, Greece; e Radiation Oncology Unit, 2nd Department of Radiology, Attikon University Hospital, Medical School, National and Kapodistrian University of Athens (NKUOA), Athens, Greece.

**Keywords:** adaptive radiotherapy (ART), anatomic changes, clinical outcomes, dosimetric changes, head and neck cancer, intensity-modulated radiation therapy (IMRT), time of adaptation

## Abstract

**Purpose:**

The current review aims to summarize the international experience of the impact of adaptive radiotherapy on dosimetry and clinical and toxicity outcomes. Additionally, it might trigger Radiation Oncologists to use ART and evaluate whether ART improves target volume coverage and/or normal tissue sparing and, consequently, therapeutic results.

**Materials and methods:**

We conducted an electronic literature search of PubMed/MEDLINE and ScienceDirect from January 2007 to January 2023. The search adhered to the PRISMA guidelines and employed keywords such as ART, HNC, parotid gland, and target volume. Furthermore, we examined the reference lists for studies pertinent to the present review. This study included both retrospective and prospective studies that were considered for inclusion.

**Conclusion:**

ART replanning appears to be a sustainable strategy to minimize toxicity by improving normal tissue sparing. Furthermore, it can enhance target volume coverage by correctly determining the specific dose to be delivered to the tumor. In conclusion, this review confirmed that ART benefits dosimetric, clinical/therapeutic, and toxicity outcomes.

## 1. Introduction

Head and neck cancer (HNC) is one of the most common malignancies in developing countries, accounting for 5% of all cancers in both males and females, with over 800,000 new cases reported worldwide each year, and is responsible for more than 400,000 deaths annually.^[1,2]^ The incidence of HNC exhibits significant regional variations, with a higher prevalence in South and Southeast Asia and certain parts of Europe.^[3,4]^

HNC encompasses a range of cancers affecting the oral cavity, pharynx, larynx, nasal cavity, paranasal sinuses, and salivary glands.^[5]^ Key risk factors included tobacco use and alcohol consumption. However, in recent years, human papillomavirus (HPV) infection has emerged as a significant risk factor, particularly for oropharyngeal cancer.^[6–9]^ Men have a higher risk of developing HNC than women, and the likelihood of diagnosis increases with age, predominantly affecting individuals over the age of 40 years. Treatment approaches for HNC typically include surgery, radiation therapy (RT), chemotherapy, or a combination of these methods, with a specific treatment plan tailored based on the cancer stage and location.^[10]^

Survival rates vary based on several factors, mainly the stage at diagnosis and tumor location; however, the 5-year survival rate is generally approximately 60%.^[11]^

## 2. Treatment evolution and current challenges of HNC RT

Most studies and statistics show that more than 65% of patients with HNC have locoregionally advanced disease at diagnosis.^[12]^ Chemoradiotherapy with systemic administration of a platinum-based regimen in combination with fractionated radiotherapy at a dose of 63 to 70 Gy is the standard of care for locoregional organ-sparing therapy for head and neck SCC. Modern radiotherapy uses imaging techniques, such as computed tomography, during treatment to accurately deliver high curative radiation doses to tumors.^[13]^ The transition from 2-dimensional conventional radiotherapy (2D-RT) to 3-dimensional conformal radiotherapy (3D-CRT) and intensity-modulated radiation therapy (IMRT) has contributed the most to achieving maximal therapeutic effects.^[14]^

In addition to the notable positive therapeutic outcomes, the issue of acute adverse effects stemming from chemoradiotherapy regimens for HNC remains significant. Chemotherapy, RT, and their combination can induce various acute and delayed adverse effects such as weight loss and anxiety. Notable acute toxicities resulting from radiation include severe mucositis, dermatitis, xerostomia, and dysphagia, some of which may necessitate the use of feeding tubes. Substantial late or chronic toxicities include xerostomia, dysphagia, and fatigue, which persistently affect the quality of life for years after treatment.^[15,16]^

### 2.1. Radiotherapy planning

Over the past 20 years, the standard of care for radiotherapy in HNC has transitioned from 2D-RT to 3D-CRT and eventually to IMRT.^[17]^ Initially, conventional radiotherapy approaches were rigid, delivering a fixed dose to the tumor and surrounding tissues. IMRT has demonstrated its superiority with its ability to decrease adverse effects on normal tissues, such as reducing xerostomia without compromising the therapeutic dose of radiation to the target volumes of both the primary and lymph nodes.^[18]^ However, because of the unique features of IMRT, such as inverse planning procedures, any anatomical changes in patients resulting from weight loss or tumor shrinkage can substantially affect the delivered radiation dose. Therefore, the ART concept was introduced, offering improved precision, reduced toxicity, and improved patient outcomes.^[19,20]^

Additional advancements in contemporary radiotherapy methods include IMRT, volumetric-modulated arc therapy (VMAT), stereotactic body radiotherapy (SBRT), image-guided radiotherapy (IGRT), and utilization of protons or heavy ions.^[19,21]^

Until the late 1990s, the standard approach for 3D-CRT involved using 6 MV photon beams and employing the 3-field method. This technique uses 2 opposing lateral fields to target the primary tumor and cervical lymph nodes in the upper and lower neck regions, respectively. A third anterior field was used to irradiate the supraclavicular lymph nodes. The lateral and anterior fields share the same isocenter to ensure precise radiation delivery. These fields were carefully aligned at the isocenter level to avoid overlapping with the radiation fields at the junction line. Periodic adjustments of the junction line during treatment are essential to facilitate a gradual transition in the dose distribution and minimize potential adverse effects. This 3-field technique involved several sequential dose boosts associated with specific prescription doses.^[20,22]^

In contrast to the “older” 3-field treatment approach, IMRT employs varying beam intensities and segments to deliver radiation that conforms to the shape of the tumor and results in a steep dose drop-off at the tumor-normal tissue boundary. IMRT is well suited for HNC because of its complex tumor geometry, proximity to critical structures, and minimal organ movement. The 2 standard IMRT planning techniques for HNC are split- and extended-field IMRT. The split-field approach treats the primary tumor and the upper neck with IMRT using a conventional anterior field in the lower neck and supraclavicular regions. Extended-field IMRT treats all tumor volumes simultaneously, but requires attention to avoid overdosing of the larynx. To treat bilateral tumors, a standard setup involves 9 coplanar 6 MV photon beams evenly positioned around the patient at angles of 0°, 40°, 80°, 120°, 160°, 200°, 240°, 280°, and 320°. For unilateral tumors, 7 coplanar beams angled from the side of the tumor were used. Care was taken to avoid directing the radiation laterally and, when necessary, to slightly adjust the gantry angle to prevent the beam from passing through the shoulder. The isocenter of the treatment is typically chosen at the central point within the area to be irradiated, given that head and neck treatments often involve relatively large treatment fields.

The delineation of target volumes is further improved by functional imaging and fusion of 18F-fluorodeoxyglucose positron-emission tomography (18FDG-PET) scans with CT scans, although variability in delineation remains a challenge. IMRT planning has transitioned from manual planning to computer-based inverse planning, which offers better dose distribution to tumor volumes and improved sparing of critical structures.^[23–26]^

VMAT, an advanced version of IMRT, further improves treatment by optimizing the treatment delivery. In traditional IMRT, the treatment plan involves numerous small radiation fields created using MLC. This can be achieved by sequentially configuring the MLC leaves to complete specific fields, administering radiation (step-and-shoot method), or continuously adjusting the MLC leaves. Simultaneously, the radiation beam was active (sliding window method). In contrast, VMAT allows for the simultaneous movement of the MLC and gantry, adjusting factors such as MLC leaf speed, gantry speed, and dose rate in real time during treatment. VMAT has gained popularity for the treatment of HNCs because of the intricate anatomy of this region. A typical VMAT plan involves 2 to 3 complete or partial arcs, depending on whether the treatment targets bilateral or unilateral areas. VMAT plans incorporate more beam angles, resulting in a more precise dose distribution within the target area than that of traditional IMRT plans. VMAT plans maintain comparable target coverage to fixed-gantry IMRT but offer enhanced uniformity. Notably, the delivery time for VMAT plans is significantly shorter (approximately 5 min), whereas fixed-gantry IMRT plans typically require 10 to 15 minutes. A comparison of the dose distribution between the 2-arc VMAT plan and 9-beam IMRT plan for patients with laryngeal cancer and bilateral cervical lymph node involvement resulted in that VMAT and IMRT effectively covered planning target volume (PTV) with comparable organ-at-risk (OAR) sparing. Occasionally, VMAT may enhance the sparing of contralateral organs at risk (OARs).^[19,27,28]^

### 2.2. Fractionation

Radiation treatment schedules for HNC are not universally standardized. Typically, standard RT refers to daily doses of 1.8 to 2 Gy, given 5 days a week in 33 to 35 fractions, delivering a therapeutic dose of 70 Gy. However, different radiation schedules have been developed to enhance the outcomes of HNC treatments. Over the past 20 years, 2 primary altered fractionation schedules, hyperfractionation and accelerated fractionation, have been extensively studied.

Hyperfractionation typically entails administering radiation at more frequent but smaller daily doses, often maintaining or slightly reducing overall treatment duration. This strategy results in a more significant biologically effective dose to tumors while improving the tolerance of late-responding normal tissues. Multiple randomized trials have indicated that hyperfractionation notably enhances tumor control in local and regional contexts, ultimately improving survival rates compared to conventional fractionation. It is essential to acknowledge that hyperfractionation may lead to more pronounced acute mucositis; however, late complications remained consistent with observations from the traditional fractionation schedules.

Accelerated fractionation involves decreasing the total treatment duration, while maintaining the number of dose fractions, total dose, and individual fraction sizes at similar or slightly reduced levels. The underlying principle of accelerated fractionation is to limit the time available for tumor cells to regenerate or repopulate during the treatment period by shortening its duration. Clinical studies of accelerated fractionation have shown notable enhancements in local and regional control and survival rates. Although these accelerated schedules may lead to intense acute mucositis, there is no increase in the risk of late complications.

For both hyperfractionation and accelerated fractionation regimens, it was essential to maintain a minimum interval of 4.5 hours between fractions. This allows sufficient time for normal tissues to undergo repair following a sublethal radiation injury. Fractionation in radiotherapy is a critical factor significantly affecting treatment effectiveness.^[19,29–32]^

In a meta-analysis conducted by Bourhis et al,^[33]^ which included 15 trials that compared conventional radiotherapy with hyperfractionation and accelerated radiotherapy in patients with squamous cell carcinoma (SCC), the findings suggested that modified fractionation radiotherapy resulted in superior tumor control and enhanced survival compared with conventional therapy. Furthermore, research conducted by Fu et al reported that, for locally advanced cancer, both hyperfractionation and accelerated fractionation were more efficacious than conventional fractionation.^[34,35]^

### 2.3. Daily imaging

Modern RT requires frequent imaging, which is necessary to improve treatment precision and outcome. Daily imaging is essential for several reasons, particularly during ART for HNC.

1. **Anatomical Variability:** The head and neck region experiences significant anatomical changes, including weight loss, tumor regression, and patient positioning shifts. Daily imaging, often via cone-beam computed tomography (CBCT), detects these changes and ensures precise tumor targeting, while sparing critical structures.
2. **Patient Positioning:** Accurate patient setup is essential for effective RT. Daily imaging verifies patient positioning and minimizes the risk of radiation toxicity to the normal tissues.
3. **Dose Escalation:** Daily imaging enables safe dose escalation by adapting treatment plans to the current anatomy. This can improve the local control and overall treatment outcomes.
4. **Reduced Margins:** Smaller treatment margins, made possible by daily imaging, minimize radiation exposure to nearby healthy tissues, crucial in the head and neck region, where critical structures are close to the tumor.
5. **Volume Shrinkage:** All studies reported varying degrees of volume shrinkage in the target structures, such as gross tumor volume (GTV), clinical target volume (CTV), and PTV. The extent of volume reduction ranged from approximately 11% to over 80%, with differences likely related to individual patient responses and tumor characteristics.

HNC poses a significant challenge in the field of RT because of its complex and dynamic anatomical structures, proximity to critical organs, and interfractional variations in tumors and surrounding healthy tissues.^[36]^ However, in contemporary practice for HNC RT, it is often common for radiation oncologists to perform treatment plans without accounting for the actual anatomical changes that may result from swelling of the irradiated areas, daily normal tissue volume alterations, tumor shrinkage, and loss of body mass and weight.^[18,19]^ All these changes can displace the target and OARs from their original positions, affecting the accuracy of the radiation dose delivered without avoiding and protecting the surrounding healthy tissues. Progress in image-guided RT has shown that volumetric changes in target volumes and OARs are often observed during IMRT in patients with HNC.^[19]^ Moreover, these changes may result in an ill-fitting immobilization mask.^[13]^

Although weight loss or a reduction in nodular volume may be apparent on physical examination, these changes have resulted in unintended (or at the very least unmonitored) deviations from the initial planning geometry. CTV undercoverage and/or organ-at-risk (OAR) overdosing may occur despite the application of isocentric image-guided alignment.^[37–39]^

## 3. The purpose of the current review

Adaptive radiotherapy (ART), which can dynamically adjust the treatment plan in response to the patient’s anatomical changes and deviations, has emerged as a promising solution to enhance target volume coverage in terms of tumor size, shape, and position, guaranteeing that the tumor receives the intended radiation dose, minimizing radiation-induced toxicity, and improving the patient’s quality of life during and after treatment. Additionally, ART offers the opportunity for optimal dose escalation when clinically indicated, potentially increasing the likelihood of tumor control without increasing the risk of damage to healthy tissues. Furthermore, ART can minimize treatment interruptions by quickly adjusting the treatment plan to accommodate sudden changes in anatomy such as those caused by weight loss during treatment. ART considers each patient’s unique anatomical changes and is a highly personalized approach that leads to better treatment outcomes.^[40]^ This individualized treatment can improve therapeutic outcomes and provide a more customized treatment experience.^[41,42]^

ART for HNC can typically be used to assess and correct tumor response and weight loss. Radiation oncologists are expected to use image-guided scans as the basis for treatment to enhance adaptation planning for treatment response.^[43,44]^

Therefore, ART is implemented to correct all morphological variations by actively adapting the treatment plans into 3 different timeframes: between therapy sessions (Offline Adaptive), immediately before a therapy session (Online Adaptive), and during a therapy session (Real-time Adaptive).^[45]^

Based on the abovementioned principles, the current review aims to summarize the international experience of the impact of Adaptive Radiotherapy (ART) on dosimetry and clinical and toxicity outcomes. Additionally, it might trigger Radiation Oncologists to use ART and evaluate whether ART improves target volume coverage and/or normal tissue sparing and, consequently, therapeutic results.

## 4. Methods and materials: literature search (PRISMA)

We conducted an electronic literature search of PubMed/MEDLINE and ScienceDirect from January 2007 to January 2023. The search adhered to the PRISMA guidelines and employed the following keywords: HNC; RT; 3-Dimensional Conformal RT; IMRT; Intensity-Modulated Proton Therapy (IMPT); Image-Guided RT; Adaptive RT; Response-Adapted RT; Anatomy-Adapted RT; Target Volume; OARs; Parotid Gland.

Furthermore, we examined the reference lists for studies pertinent to the present review. This study included both retrospective and prospective studies that were considered for inclusion.

Articles were selected for inclusion in the review based on their relevance to the project. The eligibility criteria for this review were as follows.

- Addressing dosimetric/anatomic changes.
- Involving studies with a participant count of 10 or more.

**Figure 1.**
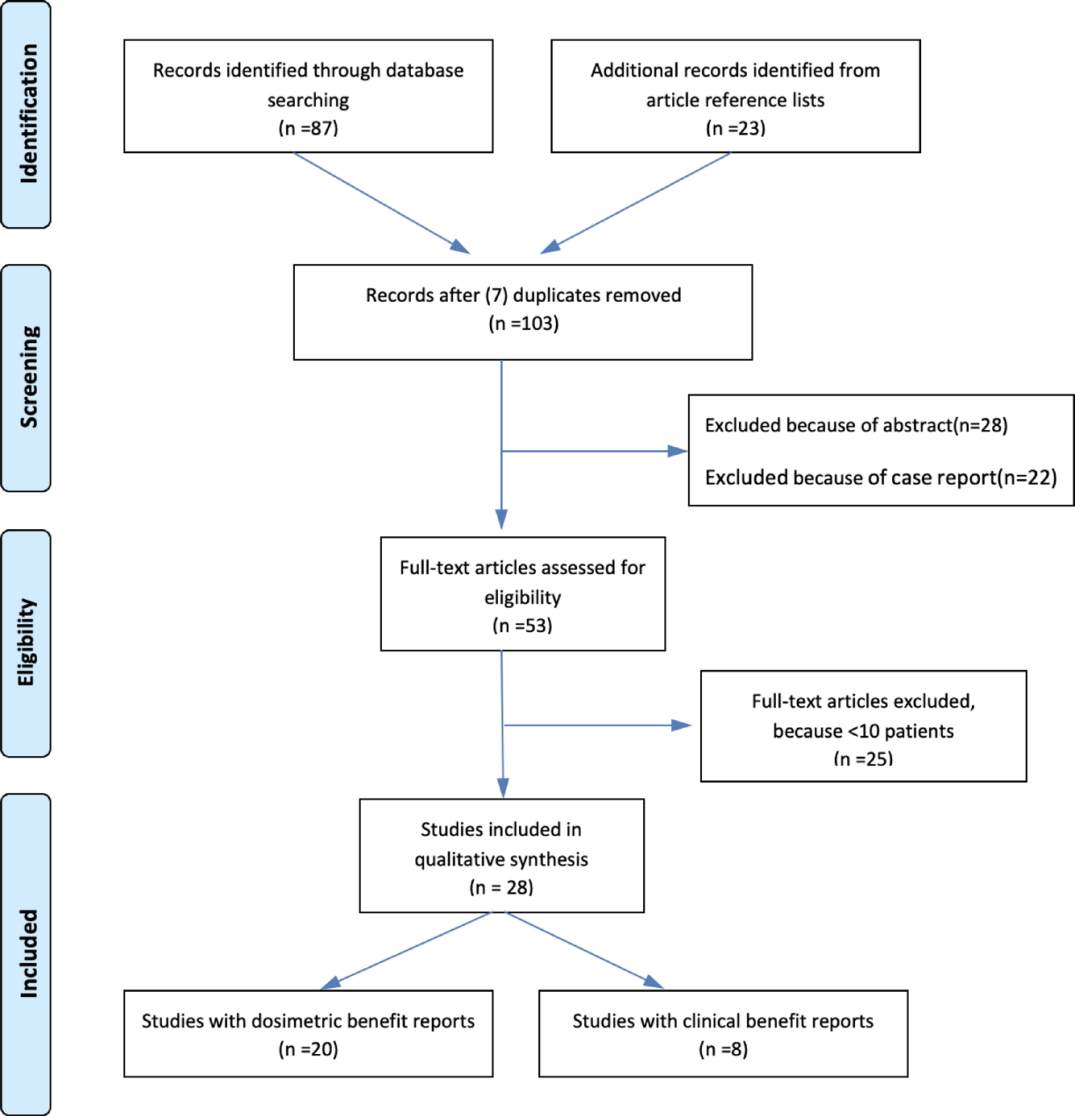
Flow chart diagram.

We thoroughly examined all incorporated studies to gather the following information: patient count, treatment methodology, frequency of replans, anatomical and dosimetric alterations, and the dosimetric and clinical advantages of ART. Studies with a restricted participant size of less than ten were excluded. Figure 1 shows the flowchart of the literature review process.

## 5. The role of ART in HNC RT (results derived from lit rev)

### 5.1. Offline and online adaptive planning

The 2 primary approaches to ART are offline and online Adaptive RT. Online adaptive planning, often called “real-time” adaptive planning, is a technique in which treatment plans are adjusted daily during the therapy. This approach is particularly relevant in situations where anatomical changes such as tumor shrinkage or variations in patient positioning occur frequently and necessitate immediate action.

Offline adaption, or “inter-fractional” adaptive planning, is suitable for systematic or slow progressive changes (e.g., tumor regression, weight loss). The treatment team can adapt based on an observed deviation in anatomy (on imaging or visible physical alterations) or follow a protocol with predefined action levels and/or surveillance scans.^[34,41,43,44,46]^

Table 1 provides an overview of the main differences between online and offline adaptive radiotherapy, highlighting their strengths and weaknesses in various clinical contexts. The choice between these approaches depends on the patient’s specific needs and the clinical circumstances.

**Table 1:** Main differences between online ART and offline ART.

Aspects	Online ART	Offline ART
Timing of Plan Adaptation	Real-time, daily or even intra-fractional	Periodic, often weekly or bi-weekly
Imaging Frequency	Daily, often using CBCT	Periodic, less frequent imaging (e.g., weekly)
Adaptation Response Time	Immediate	Delayed, typically days to weeks
Treatment Efficiency	Time-consuming, may extend treatment duration	Less time-consuming, may minimize treatment interruptions
Anatomical Changes	Continuously monitored	Assessed periodically
Resource Requirements	Requires significant resources, including advanced imaging equipment	Requires fewer resources, often relies on conventional imaging
Precision in Tumor Targeting	High, allowing rapid response to changes	Good, with adaptations at intervals
Patient Comfort and Experience	Potential for longer treatment times	Potential for more consistent treatment times
Handling of Clinical Uncertainties	Offers real-time adaptation to uncertainties	May not address uncertainties as quickly
Clinical Applications	Applicable in dynamic or rapidly changing clinical scenarios	Suited for more stable anatomical conditions

The frequency of CT scans for replanning therapy is a critical consideration in maximizing the benefits of this adaptive approach. The timing and frequency of CT scans for replanning HNC treatment vary, with some advocating daily scans. In contrast, other studies performed scans based on patient responses and clinical protocols. Typically, these scans occur at critical points during treatment, such as the start and specific intervals, to assess anatomical changes and adjust treatment plans. The goal is to precisely target tumors while minimizing the side effects and radiation exposure to healthy structures.^[47]^

### 5.2. Dosimetric considerations

Several studies have collectively investigated the dosimetric benefits of ART in the treatment of HNCs. These studies used various imaging methods, evaluation timings, and replanning frequencies. The expected dosimetric outcomes include volume shrinkage in the target structures and changes in radiation doses to OARs.^[37,38,48–60]^

### 5.3. Volume shrinkage

All studies reported varying degrees of volume shrinkage in the target structures, such as GTV, CTV, and PTV. The extent of volume reduction ranged from approximately 11% to over 80%, with differences likely related to individual patient responses and tumor characteristics.

### 5.4. Dose improvement of OARs

Multiple studies have demonstrated improvements in radiation doses to OARs, particularly to the parotid glands. The dose reduction in the parotid glands ranged from approximately 3% to > 25%, reducing xerostomia and other adverse effects. The spinal cord and brainstem doses were also lowered, enhancing the treatment safety.

### 5.5. Replanning strategies

These studies used various ART strategies, including daily CBCT, weekly CT scans, and different evaluation timing. Several studies have used multiple replicates to demonstrate ART adaptability. One study applied an online ART approach that offered real-time adaptability.

### 5.6. QA – quality assurance

Ensuring the accuracy and reliability of ART is paramount. Comprehensive quality assurance procedures, including imaging, contouring, plan adaptation, and dose calculation, are essential for maintaining ART integrity. Rigorous QA programs help to guarantee safe and effective treatment delivery.^[47]^

Table 2 summarizes the dosimetric benefits of online ART in terms of target coverage and sparing of critical OARs in various replanning strategies for HNC patients.

**Table 2:** Dosimetric benefits of ART in target covering and OAR’s sparing.

Author (yr)	Pts No		Replanning strategies				Dosimetric benefits			
			Total Dose (Gy)	Imaging method	Time of evaluation	No of replanning	Volume shrinkage	Parotid (Dmean/V26)	Spinal cord/brainstem (Dmax)	Benefit from ART
Zhao (2011)^[61]^	33		37.5 Gy (20–50 Gy)	CT	1st at 15th (±5) fr 2nd at 12th (± 4) fr 3rd after 15th fr	1	GTVp: −13.9%; GTVn: −71.9%; CTV: −3.5%;	Decreased mean dose (*P* < .05)	NR	Decreased PG dose
Capelle (2012)^[48]^	20		66 Gy (54–60 nodes; 66 Gy primary)	CT	15th fr	1	Median Volume Loss: PTV60/66 = −16% (0–45%); PTV54 = - 6.8 (−1.2–19%); GTV = −28.8% (−1.6–60%); CTV60 = − 4.1% (−0.1–10%); PG = 17.5% (−1–46%)	CohA Adjuvant CRT: PG: Dmean = −1.2 Gy/V26 = −6.3% CohB Definitive CRT: PG: Dmean = −1.2 Gy/V26 = −6.3%	SC: Dmax = 1.2 Gy	15/23 Pts improved TV dose coverage
Duma (2012)^[62]^	11		64 postop 70 radicals	MVCT	16th fr (9th–21rst)	1	NR	PG: no variation of dose	SC: −0.14 Gy	–
Jensen (2012)^[63]^	72 15 replanned		70.4	Weekly CT	Weekly	2–4	NR	CPG: −11.5%	NR	8% Improvement of coverage
Schwartz (2013)^[64]^	22		70		16^th^ and 22^nd^ Fr	1–2	NR	−0.7 Gy	NR	Increase coverage and dose homogeneity
Bhandari (2014)^[21]^	15		NR	CT	3rd week of treatment Between 18th and 20th fr	1 (HP vs AP)	Mean Volume Loss: GTV = −44.32 cc; CTV = 82.2 cc; PTV = −149.83 cc	RPG Dmean = +5.56 ± 4.99 Gy (*P* < .04); LPG Dmean =+3.28 ± 3.32 Gy (*P* < .003);	SC Dmax = +1.25 ± 2.14 (*P* = .04) BS Dmax = +3.88 ± 3.22 (*P* < .02)	TV and OARs
Lu J (2014)^[49]^	12		66 to Primary GTV (D95)	CT	25th fr	1 (HP vs AP)	Mean Volume Change: PGTV = −16.4 ± 27.3; PTV1 = +3.8 ± 6.3; PTV2 = −8.8 ± 12.0 cc; rPG = −24.6 ± 11.9; IPG = −35.1 ± 20.1	RPG = −24.6 ± 11.9; LPG = −35.1 ± 20.1	SC and BS: 8/12 No-ART Pts exceeded the constraint without replanning	TV and OARs
Olteanu (2014)^[50]^	10		70.2 Gy	PET-CT	10th, 20th and last day of treatm for dose sum	2 (11th–20th fr and 21st–30th)	Reduced GTV volumes (18.6–93.3%) after 18th fr	PG: reduced median dose 4.6–7.1% (*P* > .05)	OARs: dose-differences from −7.1 to 7.1%	TV and OARs
Reali (2014)^[51]^	10		68.4–70.2 Gy	CT	Weeks 3, 5, and 7 of treatment	1	PTVs mean relative shift = 0.1 cm (not taken in consideration for replan); PG volume decreased mainly for Ipsilateral PG	NR	SC mean relative shift = 0 cm	PGs
Castelli (2015)^[52]^	15		70 Gy	Weekly CT	Weekly	Weekly replanning	CTV70 decreased by a mean value of 31% (ranging 3% to − 13%) PG volumes decreased by a mean value of 28.3% (ranging from 0.0% to 63.4%)	PG Dmean: -4.8 Gy (67% Pts) and −3.9 Gy (33% Pts) Median contralateral PG dose-decrease: −2.0 Gy. (from 27.9 to 25.9 Gy)	NR	PG reduced dose
Zhang (2016)^[53]^	13		70	Weekly CT	Weekly	6 weekly replans	Mean Volume Reduction CTV70 = 24.43%	With standard IMRT: Mean PG overdose of 4.1 Gy vs planned dose. In Replan: PG mean dose −3.1 Gy compared planned dose	NR	PG Benefit > 4 Gy (34% one PG – 15% both PGs)
Van Kranen (2016)^[54]^	19		70	Daily CBCT	10th fr	1 (10th fr or max in 2nd week of treat)	In 5th week: decreased CTVn & CTVnboost < −10%	Margin reduction and improvement in OAR dose ≈ 1 Gy/mm	NR	PGs: improved D99% with - at least - 3 Gy
Dewan (2016)^[55]^	30		GTV = 70 Gy; CTV = 66 Gy; PTV = 60 Gy	Weekly CT	20th fr	1 (+ 1 hybrid)	Shrinkage: GTV: 47.62%; CTV: 43.76%; PTV: 39.69%. IPG: 33.65%; CPG: 31.06%	Mean dose to ipsilateral parotid was significantly reduced with replanning by 26.04 + 29.14 % (*P* = .001).	SC: Replanning reduced mean Dmax, D2% and D1% by 28.26 + 10.27%, 30.87 + 12.83% and 31.20 + 13.09% respectively as compared to delivered dose (*P* < .01)	TV and OARs
Deng (2017)^[56]^	20		66–71.6 Gy	CT	5th and 15th frs	2 replans + 2 hybrid replans (5th and 15th fr)	Increase in V95; PTVnx: Decreased Vmax and V110	Left PGs: Dmean = 3.67 Gy; V30 = 3.66 Gy	In ART plans: SC: Dmax decreased by 2.42 Gy; BS: Dmax decreased by 2.42 Gy	TV and OARs
Surucu (2017)^[19]^	51		70.2 Gy	CT and CBCT	After median dose of 37.8 Gy (27–48.6)	1 (in 34/51 pts)	Median TVRR: 35.2% (−18.8–79.6%)	IPG Dmean = – 6.2%; CPG Dmean = −2.5%;	SC Dmax = −4.5% BS Dmax = −3.0%	TV and OARs
Castelli (2018)^[57]^	37	70 Gy		Weekly CT	Weekly	Weekly replanning	NR	Median contralateral PG dose-decrease: −2.0 Gy. (from 27.9 to 25.9 Gy)	NR	PGs: reduced dose in 89% of Pts; CTV: increased dose in 67% of Pts; Both benefits: in 56% of Pts
Aly (2019)^[20]^	10	70 Gy		CT	Mainly in 10th fr and 15th fr	Mean 2 (range 0–5)	Volume Reductions: GTVp = 25%; CTVp = 18%; GTVn = 44%; CTVn = 28%; PTV70 = 11%; PTV60 no significant change	PG volume decrease 28% during treatment	NR	Improved coverage of PTV70 and PTV60
Hay (2020)^[59]^	20	65 Gy		CT and CBCT	At fraction 16th	1 (19th fr)	NR	NR	SC: 3 Pts exceed max dose without replan	Benefit in OARs
Mnejja (2020)^[58]^	20	69.96 Gy		CT	At dose 38 Gy	1 (19th fr)	Reduction in TV: 58.56% (GTVnodes); 29.52% (GTVtumor)	NR	NR	Deterioration of Tumor Coverage in no-ART Pts
Avkshtol (2023)^[60]^	21	NR		CT and CBCT	Frequency of adapts at the discretion of treat physician	1–5 Median 13.5th and 19th fr	NR	IPG: −13.2 (node + pts) −11.24% (T3/4 disease pts) CPG: −3.78 (node + pts) −1.68 (T3/4 disease pts)	SC Dmax: −9.65 (node + pts) −9.32 (T3/4 disease pts) BS Dmax: −6.41 (node + pts) −5.38 (T3/4 disease pts)	PTV coverage PTV dose homogeneity OARs

These studies have explored the advantages of online ART, considering factors such as the total dose, imaging method, timing of evaluation, number of replanning events, volume shrinkage, and benefits to the OARs. Duma et al^[62]^ studied 11 patients and demonstrated parotid gland sparing with minimal spinal cord dose variation. Two studies underscored the significant dosimetric advantages of online ART in enhancing the target coverage for patients with HNC. Jensen et al^[63]^ conducted a comprehensive investigation of 72 patients who underwent 15 replanned events. Their findings revealed a substantial improvement in target coverage, suggesting that online ART can dynamically adapt to changes in patient anatomy and ensure more precise irradiation of the tumor volume. Similarly, Schwartz et al^[64]^ reported an increased target coverage and improved dose homogeneity in a cohort of 22 patients. These results further emphasize that online ART can adapt to anatomical variations and optimize the target coverage and dose distribution.

### 5.7. Clinical considerations

Table 3 summarizes the clinical outcomes of ART for HNC across different tumor sites, demonstrating the potential benefits of ART in improving locoregional control, survival rates, and toxicity profiles. Collectively, these studies support the utility of ART for enhancing the therapeutic landscape of patients with HNC.

**Table 3:** ART Studies - clinical outcomes.

Author (yr)	No PTS	Replanning strategies					Clinical endpoint		
	ART (NO-ART)	Tumor site	Total dose (Gy)	No of replanning	Timing (fraction)	Follow-up (mo)	Locoregional control and survival	Acute toxicity (%)	Late toxicity
	33 (66)	NPC	70	1	15th (± 5)	38	3-yrs LRFS 72.7% (ART); 68.1% (No-ART) *P* = .3	NR	Less xerostomia and mucosal with ART for N2 and N3 pts
Schwartz (2012)^[27]^	22 (0)	OPC	66–70	1 or 2	16th and 22nd	31	2-yrs LRC = 95%	G III mucositis: 100%, G II xerostomia: 55% G III xerostomia: 5%	Full preservation or functional recovery of speech and eating at 20 months
Yang (2013)^[65]^	86 (43)	NPC	70–76	1 or 2	15th and/or 25th Fr	29	2-yrs LRC = 97.2% (ART); 82.2% (No-ART) *P* = .04 2-yrs OS = 89.8% (ART); 82.2% (No-ART) *P* = .47	NR	Improvement in OoL with ART
Chen (2014)^[66]^	51 (266)	LAHNC	60 ^b^ 70 ^μ^	1	40 Gy (10–58 Gy)	30	2-yrs LRC = 88% (ART); 79% (No-ART) *P* = .01 2-yrs OS = 73% (ART); 79% (No-ART) *P* = .55	G III: 39% (ART) 30% (No-ART) *P* = .45	G III = 14% (ART) 19% (No-ART) *P* = .71
Kataria (2016)^[67]^	36 (0)	LAHNC	70	1	54 Gy	–	2-yrs DFS = 72%. 2-yrs OS = 75%	G II-III mucositis = 100%	–G II xerostomia = 8%–G II mucosal = 11%.–No G III toxicity
Surucu (2017)^[19]^	51 (17)	LAHNC	70.2	1	37.8 Gy (47–48.6)	17.6 mos	Median DFS: 14.8 (0.9–57.5) mos Median OS: 21.1 (4.5–61.4) mos Residual Disease: 11.8 % Locoregional Control: 64.7% Metastatic disease: 23.5%	Mucositis 35.3% Xerostomia NA Dysphagia 41.2%	Mucositis NA Xerostomia 3.3% Dysphagia 20%
Mostafa (2021)^[68]^	49 (0)	LAHNC	70	1	42 Gy (37–44.1)	Median 18 months (6.5–31)	1-yrs PFS 89.7%; 2-yrs PFS 70.2% 1-yrs OS 89%; 2-yrs OS 83 %	G II-III mucositis: 32.6–36.7 % G II–III Xerostomia: 44.9–16.3%	–
Zhou (2022)^[69]^	290 (147)	NPC	NA	1–2	15th-25th Fr	median (months) 104 ART; 96 no-ART	8-yrs LRFS ART 87.4%; No-ART 75.6% 8-yrs DMFS ART 82.3%; No-ART 76.8% OS: 60.9 % (ART)/ 59.4 % (no-ART)	NR	G I–II Xerostomia: ART 96.5% - no-ART 90.5% G III–IV Xerostomia: ART 3.5% - no-ART 9.5%

ART = adaptive, disease-free survivalOS = fractionLRC = LAHNClocally advanced head and neck cancer, loco-regional controlLRFS = loco-regional freesurvivalDFS = Nbnumber, Nb pts = number of patients, NPC = nasopharyngeal carcinoma, OPC = oropharynx cancer, radiotherapyFr = overall survival.

Two fundamental studies explained the potential benefits of ART in improving clinical outcomes in patients with nasopharyngeal cancer (NPC). Zhao et al^[61]^ evaluated clinical results in a cohort of 33 patients who received ART and 66 patients who underwent non-ART treatment. Their results indicated a 3-year locoregional failure-free survival (LRFS) rate of 72.7% in the ART group compared with 68.1% in the non-ART group. Notably, the ART approach showed a nonsignificant reduction in xerostomia and mucosal issues, particularly in N2 and N3 patients, highlighting the potential benefits of ART in improving patient comfort and quality of life. Yang et al^[65]^ conducted a study involving 86 patients with NPC, of whom 43 received non-ART treatment. Their findings demonstrated significant improvements in clinical outcomes in the ART group, with a 2-year locoregional control (LRC) rate of 97.2% compared to 82.2% in the non-ART group. Additionally, the ART group’s 2-year overall survival (OS) rate was 89.8%, while the non-ART group had an OS rate of 82.2%.

Similar studies on Oropharyngeal Cancer (OPC), such as the study by Schwartz,^[64]^ examined ART in 22 patients with OPC, and their results revealed an impressive 2-year local-regional control rate of 95%. Notably, this study highlighted a favorable toxicity profile, indicating that most patients either maintained or achieved complete preservation or functional recovery of speech and eating within a relatively short period of 20 months.

In locally advanced head and neck cancer (LAHNC), 2 critical studies, Chen et al^[66]^ and Mostafa et al,^[68]^ collectively underscored the potential benefits of ART in LAHNC, showing improved disease control without a significant increase in acute toxicity, which can ultimately lead to enhanced patient survival rates. The variation in clinical endpoints between the 2 studies highlights the multifaceted nature of ART’s impact of ART on LAHNC, demonstrating that this approach can be tailored to individual patient needs while striving for improved therapeutic efficacy and quality of life.

The last category included a mixed population of patients with HNC. In a survey by Zhou et al 2022^[69]^ of a substantial cohort of 290 patients who received ART, the investigators compared their clinical outcomes to those of 147 patients who underwent non-ART treatment. The findings were particularly promising, with ART demonstrating an impressive 8-year locoregional failure-free survival (LRFS) of 87.4% compared with 75.6% in the non-ART group. Notably, ART was associated with a reduction in xerostomia, with a notably favorable rate of Grade I–II and a lower rate of Grade III–IV xerostomia compared to the non-ART approach.^[47,61,64–69]^

## 6. Conclusions

Only a relatively low number of papers regarding ART are in international literature. The studies we reported also had few patients and many varieties of RT techniques, schedules, treatment modalities, and replanning timing. Moreover, a global consensus in ART practice is lacking worldwide.^[70]^ All the above are our review’s most significant limiting conditions, which prevented us from doing comparative statistical analysis in our study.

Nevertheless, our review reveals a comprehensive and evolving landscape of adaptive radiotherapy for HNC, with the primary goal of enhancing local control while maintaining the patient’s quality of life.

Adaptive radiotherapy, with its ability to modify treatment plans in response to anatomical changes during therapy, has shown great promise for improving therapeutic outcomes in patients with HNC. Studies have consistently demonstrated that adaptive radiotherapy provides better target volume coverage and conformality, resulting in enhanced dose delivery to the tumor while minimizing radiation exposure to adjacent healthy tissues. This is particularly critical in HNC treatment, where precision is vital for balancing therapeutic efficacy and patient well-being.

Thus, the clinical implications of adaptive radiotherapy are promising. Enhanced target coverage and reduced toxicity improved the local control and survival rates. Additionally, adapting treatment plans in real-time or periodically allows for individualized patient care, addressing the unique challenges of HNCs, such as anatomical changes due to weight loss, tumor shrinkage, or edema. Reduced radiation exposure to surrounding normal tissues results in a lower incidence of acute and chronic side effects. Patients who underwent adaptive radiotherapy experienced fewer instances of xerostomia, dysphagia, and other debilitating complications. This improves quality of life during and after treatment and enables patients to tolerate fully prescribed courses of radiotherapy.

However, the successful implementation of adaptive radiotherapy is contingent on several factors, including robust imaging techniques, skilled staff, and resource availability. Large-scale prospective clinical trials are needed to solidify existing evidence and establish guidelines for the broader application of adaptive radiotherapy in HNC treatment.

In summary, adaptive radiotherapy has the potential to revolutionize HNC treatment by optimizing target volume coverage and sparing normal tissues. With its dosimetric and clinical benefits, adaptive radiotherapy is promising for enhancing the therapeutic outcomes and overall well-being of patients with HNC. Further research, technological advancements, and clinical standardization will continue to refine the role of adaptive radiotherapy in managing this challenging disease.

## Author contributions

**Data curation:** Foteini Simopoulou, Ioannis Georgakopoulos, Christina Armpilia, Pantelis Skarlos, Vasiliki Softa.

**Investigation:** Rafaela Avgousti, Vasiliki Softa.

**Resources:** Christina Armpilia, Pantelis Skarlos.

**Supervision:** George Kyrgias, Kiki Theodorou, Vassilis Kouloulias, Anna Zygogianni.

**Writing – original draft:** Foteini Simopoulou.

**Writing – review & editing:** George Kyrgias, Ioannis Georgakopoulos.

## References

[1] Joshi P Dutta S Chaturvedi P Nair S. Head and neck cancers in developing countries. Rambam Maimonides Med J. 2014;5:e0009. 24808947 10.5041/RMMJ.10143 PMC4011474

[2] Siegel RL Miller KD Wagle NS Jemal A. Cancer statistics, 2023. CA Cancer J Clin. 2023;73:17–48. 36633525 10.3322/caac.21763

[3] Marur S Forastiere A. Head and neck squamous cell carcinoma: update on epidemiology, diagnosis, and treatment. Mayo Clin Proc. 2016;91:386–96. 26944243 10.1016/j.mayocp.2015.12.017

[4] Dhull K Atri R Dhankhar R Chauhan A Kaushal V. Major risk factors in head and neck cancer: a retrospective analysis of 12-year experiences. World J Oncol. 2018;9:80–4. 29988794 10.14740/wjon1104w PMC6031231

[5] Ferlay J Soerjomataram I Ervik M Dikshit R Eser S Mathers C. GLOBOCAN 2012 v1.0, Cancer Incidence and Mortality Worldwide: IARC Cancer Base No. 11. Lyon, France: International Agency for Research on Cancer Available at: http://globocan.iarc.fr. 2012.

[6] Gregory T Scott M George E Waun K. Head and neck cancer. In: James FH Emil FN Robert CB Donald WK Donald LM Ralph RW, (eds). Cancer Medicine. Philadelphia: Lea and Febiger. 1993:1211–1274.

[7] Cogliano V Baan R Straif K. Preventable exposures associated with human cancers. J Natl Cancer Inst. 2011;103:1827–39. 22158127 10.1093/jnci/djr483 PMC3243677

[8] Zygogianni A Kyrgias G Karakitsos P. Oral squamous cell cancer: early detection and the role of alcohol and smoking. Head Neck Oncol. 2011;3. 10.1186/1758-3284-3-2 PMC3022893 21211041

[9] Zygogianni A Kyrgias G Mystakidou K. Potential role of the alcohol and smoking in the squamous-cell carcinoma of the head and neck: review of the current literature and new perspectives. Asian Pac J Cancer. 2011;12:339–44. 21545191

[10] Machiels J Rene Leemans C Golusinski W. Squamous cell carcinoma of the oral cavity, larynx, oropharynx, and hypopharynx: EHNS-ESMO-ESTRO clinical practice guidelines for diagnosis, treatment, and follow-up. Ann Oncol. 2020;31:1462–75. 33239190 10.1016/j.annonc.2020.07.011

[11] Vokes E. Head and neck cancer. In: Fauci AS Braunwald E Isselbacher KJ., (eds). Harrison’s Principles of Internal Medicine. New York: McGraw Hill. 2005;16:503–506.

[12] Pai S Westra W. Molecular pathology of head and neck cancer: implications for diagnosis, prognosis, and treatment. Annu Rev Pathol. 2009;4:49–70. 18729723 10.1146/annurev.pathol.4.110807.092158 PMC3703474

[13] Mali SB. Adaptive radiotherapy for head neck cancer. J Maxillofac Oral Surg. 2016;15:549–54. 27833352 10.1007/s12663-016-0881-y PMC5083691

[14] Kouloulias V Thalassinou S Platoni K. The treatment outcome and radiation-induced toxicity for patients with head and neck carcinoma in the IMRT era: a systematic review with dosimetric and clinical parameters. Biomed Res Int. 2013;401261. 10.1155/2013/401261 PMC3818806 24228247

[15] Ackerstaff A Rasch C Balm A. Five-year quality of life results of the randomized clinical phase III (RADPLAT) trial, comparing concomitant intra-arterial versus intravenous chemoradiotherapy in locally advanced head and neck cancer. Head Neck. 2012;34:974–80. 21818820 10.1002/hed.21851

[16] Langendijk J Doornaert P Verdonck-de Leeuw I Leemans C Aaronson N Slotman B. Impact of late treatment-related toxicity on quality of life among patients with head and neck cancer treated with radiotherapy. J Clin Oncol. 2008;26:3770–6. 18669465 10.1200/JCO.2007.14.6647

[17] NCCN Clinical Practice Guidelines in Oncology. Head and neck cancers, version 2.2020. J Natl Compr Canc Netw. 2020;18:873–98. 32634781 10.6004/jnccn.2020.0031

[18] Nutting C Morden J Harrington K Urbano J Bhide S. Parotid-sparing intensity-modulated versus conventional radiotherapy in head and neck cancer (PARSPORT): a phase 3 multicenter randomized controlled trial. Lancet Oncol. 2011;12:127–36. 21236730 10.1016/S1470-2045(10)70290-4 PMC3033533

[19] Shang Q Shen ZL Ward MC Joshi NP Koyfman SA Xia P. Evolution of treatment planning techniques in external-beam radiation therapy for head and neck cancer. Appl Radiat Oncol. 2015;4:18–25.

[20] Connell P Hellman S. Advances in radiotherapy and implications for the next century: a historical perspective. Cancer Res. 2009;69:383–92. 19147546 10.1158/0008-5472.CAN-07-6871

[21] de Arruda F Puri D Zhung J. Intensity-modulated radiation therapy for treating oropharyngeal carcinoma: memorial Sloan-Kettering cancer center. Int J Radiat Oncol Biol Phys. 2006;64:363–73. 15925451 10.1016/j.ijrobp.2005.03.006

[22] Mallick I Waldron J. Radiation therapy for head and neck cancer. Semin Oncol Nurs. 2009;25:193–202. 19635398 10.1016/j.soncn.2009.05.002

[23] Ghosh A Gupta S Johny D Bhosale V Negi M. One study assessed the dosimetric impact of anatomical changes in the parotid glands and tumor volume during intensity-modulated radiotherapy using Simultaneous Integrated Boost (IMRT-SIB) for head and neck squamous cell cancers. Cancer Med. 2021;10:5175–90. 34159749 10.1002/cam4.4079 PMC8335829

[24] Burela N Soni T Patni N Natarajan T. Adaptive intensity-modulated radiotherapy in head and neck cancer: a volumetric and dosimetric study. J Cancer Res Ther. 2019;15:533–8. 31169216 10.4103/jcrt.JCRT_594_17

[25] Lee C Langen K Lu W. Assessment of parotid gland dose changes during radiotherapy for head and neck cancer using daily megavoltage computed tomography and deformable image registration. Int J Radiat Oncol Biol Phys. 2008;71:1563–71. 18538505 10.1016/j.ijrobp.2008.04.013

[26] Thomson D Beasley W Garcez K. Relative plan robustness of step-and-shoot vs. rotational intensity-modulated radiotherapy on repeat computed tomography simulation for weight loss in head and neck cancer. Med Dosim. 2016;41:154–8. 26993081 10.1016/j.meddos.2016.01.001

[27] Buciuman N Marcu L. Adaptive radiotherapy in head and neck cancer using volumetric modulated arc therapy. Am J Pers Med. 2022;12:668. 10.3390/jpm12050668 PMC9143588 35629090

[28] Nicosia L Sicignano G Rigo M. Daily dosimetric variation between image-guided volumetric modulated arc radiotherapy and MR-guided daily adaptive radiotherapy for stereotactic body radiotherapy in prostate cancer. Acta Oncol. 2021;60:215–21. 32945701 10.1080/0284186X.2020.1821090

[29] Barker J Garden A Ang K. Quantifying volumetric and geometric changes occurring during fractionated radiotherapy for head and neck cancer using an integrated CT/linear accelerator system. Int J Radiat Oncol Biol Phys. 2004;59:960–70. 15234029 10.1016/j.ijrobp.2003.12.024

[30] Abdelhafiz N Mahmoud D Gad M Essa H Morsy A. Effect of definitive hypofractionated radiotherapy concurrent with weekly cisplatin treatment on locally advanced squamous cell carcinoma of the head and neck. J Med Life. 2023;16:743–50. 37520484 10.25122/jml-2023-0003 PMC10375354

[31] Baujat B Borhis J Blanchard P. Hyperfractionated or accelerated radiotherapy for head and neck cancer. Cochrane Database Syst Rev. 2010;2010:CD002026. 21154350 10.1002/14651858.CD002026.pub2 PMC8407183

[32] Lacas B Bourhis J Overgaard J. MARCH Collaborative Group. Role of radiotherapy fractionation in head and neck cancers (MARCH): an updated meta-analysis. Lancet Oncol. 2017;18:1221–37. 28757375 10.1016/S1470-2045(17)30458-8 PMC5737765

[33] Bourhis J Overgaard J Audry H. Meta-Analysis of Radiotherapy in Carcinomas of Head and neck (MARCH) Collaborative Group. Hyperfractionated or accelerated radiotherapy in head and neck cancer: a meta-analysis. Lancet. 2006;368:843–54. 16950362 10.1016/S0140-6736(06)69121-6

[34] Bertholet J Anastasi G Noble D. Patterns of practice for adaptive and real-time radiation therapy (POP-ART RT): part II offline and online plan adaptation for interfractional changes. Radiother Oncol. 2020;153:88–96. 32579998 10.1016/j.radonc.2020.06.017 PMC7758781

[35] Fu K Pajak T Trotti A. A Radiation Therapy Oncology Group (RTOG) phase III randomized study to compare hyperfractionation and two variants of accelerated fractionation to standard fractionation radiotherapy for head and neck squamous cell carcinomas: first report of RTOG 9003. Int J Radiat Oncol Biol Phys. 2000;48:7–16. 10924966 10.1016/s0360-3016(00)00663-5

[36] Yeh SA. Radiotherapy for head and neck cancer. Semin Plast Surg. 2010;24:127–36. 22550433 10.1055/s-0030-1255330 PMC3324252

[37] Aly F Miller A Jameson M. A prospective study of weekly intensity modulated radiation therapy plan adaptation for head and neck cancer: improved target coverage and organ at risk sparing. Australas Phys Eng Sci Med. 2019;42:43–51. 30406923 10.1007/s13246-018-0707-y

[38] Bhandari V Patel P Gurjar O Lal Gupta K. Impact of repeat computerized tomography replan in the radiation therapy of head and neck cancers. J Med Phys. 2014;39:164–8. 25190995 10.4103/0971-6203.139005 PMC4154184

[39] Mahmoud O Reis I Samuels M. Prospective pilot study comparing the need for adaptive radiotherapy in unresected bulky disease and postoperative patients with head and neck cancer. Technol Cancer Res Treat. 2017;16:1014–21. 28671024 10.1177/1533034617717624 PMC5762062

[40] Glide-Hurst C Lee P Yock A. Adaptive radiation therapy (ART) strategies and technical considerations: a state of the ART review from NRG oncology. Int J Radiat Oncol Biol Phys. 2021;109:1054–75. 33470210 10.1016/j.ijrobp.2020.10.021 PMC8290862

[41] Heukelom J Fuller C. Head and neck cancer Adaptive Radiation Therapy (ART): conceptual considerations for the informed clinician. Semin Radiat Oncol. 2019;29:258–73. 31027643 10.1016/j.semradonc.2019.02.008 PMC6559245

[42] Alfouzan AF. Radiation therapy in head and neck cancer. Saudi Med J. 2021;42:247–54. 33632902 10.15537/smj.2021.42.3.20210660 PMC7989258

[43] Figen M Oksuz D Duman E. Radiotherapy for head and neck cancer: evaluation of triggered adaptive replanning in routine practice. Front Oncol. 2020;10:579917. 33282734 10.3389/fonc.2020.579917 PMC7690320

[44] Nasser N Yang G Koo J. Head and neck treatment planning strategy for a CBCT-guided ring-gantry online adaptive radiotherapy system. J Appl Clin Med Phys. 2023:e14134. 37621133 10.1002/acm2.14134 PMC10691641

[45] Schwartz DL Garden AS Thomas J. Adaptive radiotherapy for head-and-neck cancer: initial clinical outcomes from a prospective trial. Int J Radiat Oncol Biol Phys. 2012;83:986–93. 22138459 10.1016/j.ijrobp.2011.08.017 PMC4271827

[46] Bobić M Lalonde A Nesteruk K. Large anatomical changes in head and neck cancers: dosimetric comparison of online and offline adaptive proton therapy. Clin Transl Radiat Oncol. 2023;40:100625. 37090849 10.1016/j.ctro.2023.100625 PMC10120292

[47] Surucu M Shah K Roeske J Choi M Small W Emami B. Adaptive radiotherapy for head and neck cancer. Technol Cancer Res Treat. 2017;16:218–23. 27502958 10.1177/1533034616662165 PMC5616033

[48] Capelle L Mackenzie M Field C Parliament M Ghosh S Scrimger R. Adaptive radiotherapy using helical tomotherapy for head and neck cancer in definitive and postoperative settings: initial results. Clin Oncol (R Coll Radiol). 2012;24:208–15. 22196796 10.1016/j.clon.2011.11.005

[49] Lu J Ma Y Chen J. Assessment of anatomical and dosimetric changes using a deformable registration method during intensity-modulated radiotherapy for nasopharyngeal carcinoma. J Radiat Res. 2014;55:97–104. 23728319 10.1093/jrr/rrt076 PMC3885110

[50] Olteanu L Berwoutz D Madani I. Comparative dosimetry of three-phase adaptive and non-adaptive dose-painting IMRT for head and neck cancer*s*. Radiother Oncol. 2014;111:348–53. 24746575 10.1016/j.radonc.2014.02.017

[51] Reali A Anglesio S Mortellaro G. Volumetric and positional changes in planning target volumes and organs at risk using computed tomography imaging during intensity-modulated radiation therapy for head–neck cancer: an ‘‘old’’ adaptive radiation therapy approach. La Radiologia Medica. 2014;119:714–20. 24510758 10.1007/s11547-014-0386-z

[52] Castelli J Simon A Louvel G. Impact of head and neck cancer adaptive radiotherapy to spare the parotid glands and decrease the risk of xerostomia. Radiat Oncol. 2015;10:6. 25573091 10.1186/s13014-014-0318-z PMC4311461

[53] Zhang P Simon A Rigaud B Castelli J Ospina Arango J. Optimal adaptive IMRT strategy to spare the parotid glands in oropharyngeal cancer. Radiother Oncol. 2016;120:41–7. 27372223 10.1016/j.radonc.2016.05.028

[54] van Kranen S Hamming-Vrieze O Wolf A Damen E van Herk M Sonke J-J. Head and neck margin reduction with adaptive radiation therapy: treatment plan robustness against anatomical changes. Int J Radiat Oncol Biol Phys. 2016;96:653–60. 27681762 10.1016/j.ijrobp.2016.07.011

[55] Dewan A Sharma S Dewan A. Impact of adaptive radiotherapy on locally advanced head and neck cancer - a dosimetric and volumetric study. Asian Pac J Cancer Prev. 2016;17:985–92. 27039824 10.7314/apjcp.2016.17.3.985

[56] Deng S Liu X Lu H Huang H Shu L. Three-phase adaptive radiation therapy for patients with nasopharyngeal carcinoma undergoing intensity-modulated radiation therapy: dosimetric analysis. Technol Cancer Res Treat. 2017;16:910–16. 28511585 10.1177/1533034617709396 PMC5762048

[57] Castelli J Simon A Laford C. Adaptive radiotherapy for head and neck cancer. Acta Oncol. 2018;57:1284–92. 30289291 10.1080/0284186X.2018.1505053

[58] Mnejja W Daoud H Fourati N. Dosimetric impact on changes in target volume during intensity-modulated radiotherapy for nasopharyngeal carcinoma. Rep Pract Oncol Radiother. 2020;25:41–5. 31889919 10.1016/j.rpor.2019.12.012 PMC6931189

[59] Hay L Paterson C McLoone P Miguel-Chumacero E Valentine R. Dose analysis using CBCT and synthetic CT during head and neck radiotherapy: a single-center feasibility study. Tech Innov Patient Support Radiat Oncol. 2020;23:21–9. 10.1016/j.tipsro.2020.02.004 PMC7093804 32226833

[60] Avkshtol V Meng B Shen C Choi B Okoroafor C. Early experience of online adaptive radiation therapy for definitive radiation of head and neck cancer patients. Adv Radiat Oncol. 2023;8:101256. 37408672 10.1016/j.adro.2023.101256 PMC10318268

[61] Zhao L Wan Q Zhou Y Deng X. Role of replanning in fractionated intensity-modulated radiotherapy for nasopharyngeal carcinoma. Radiother Oncol. 2011;98:23–7. 21040992 10.1016/j.radonc.2010.10.009

[62] Duma M Kamper S Schuster J Winkler C Geinitz H. Adaptive radiotherapy for soft tissue changes during helical tomotherapy for head and neck cancer. Strahlenther Onkol. 2012;188:243–7. 22294198 10.1007/s00066-011-0041-8

[63] Jensen A Nill S Huber P. Clinical concept of interfractional adaptive radiation therapy in the treatment of head and neck cancer. Int J Radiat Oncol Biol Phys. 2012;82:590–6. 21310549 10.1016/j.ijrobp.2010.10.072

[64] Schwartz D Garden A Shah S Chronowski G Sejpal S. Adaptive radiotherapy for head and neck cancer: dosimetric results from a prospective clinical trial. Radiother Oncol. 2013;106:80–4. 23369744 10.1016/j.radonc.2012.10.010

[65] Yang H Hu W Wang W Chen P Ding W Luo W. Replanning during intensity-modulated radiation therapy improved quality of life in patients with nasopharyngeal carcinoma. Int J Radiat Oncol Biol Phys. 2013;85:e47–54. 23122981 10.1016/j.ijrobp.2012.09.033

[66] Chen A Daly M Cui J. Clinical outcomes of patients with head and neck cancer treated with intensity-modulated radiotherapy with and without adaptive replanning. Head Neck. 2014;36:1541–6. 23996502 10.1002/hed.23477

[67] Kataria T Gupta D Goyal S. Clinical outcomes of adaptive radiotherapy for head and neck cancer. Br J Radiol. 2016;89:20160085. 26986461 10.1259/bjr.20160085 PMC5258178

[68] El-Shahat M Elshishtawy W Al-Agamawi A. Evaluation of adaptive radiation therapy in treatment of locally advanced head and neck cancers. Am Al-Azhar Intern Med J. 2021;2:51–8.

[69] Zhou X Wang W Zhou C. Long-term outcomes of replanning during intensity-modulated radiation therapy in patients with nasopharyngeal carcinoma: an updated and expanded retrospective analysis. Radiother Oncol. 2022;170:136–42. 35288229 10.1016/j.radonc.2022.03.007

[70] Lee V Schetllno G Nisbet A. UK adaptive radiotherapy practices for head and neck cancer patients. BJR Open. 2020;2:20200051. 33367201 10.1259/bjro.20200051 PMC7749087

